# Dealing with substantial heterogeneity in Cochrane reviews. Cross-sectional study

**DOI:** 10.1186/1471-2288-11-22

**Published:** 2011-02-24

**Authors:** Jeppe B Schroll, Rasmus Moustgaard, Peter C Gøtzsche

**Affiliations:** 1 Nordic Cochrane Centre, Rigshospitalet and University of Copenhagen, Denmark

## Abstract

**Background:**

Dealing with heterogeneity in meta-analyses is often tricky, and there is only limited advice for authors on what to do. We investigated how authors addressed different degrees of heterogeneity, in particular whether they used a fixed effect model, which assumes that all the included studies are estimating the same true effect, or a random effects model where this is not assumed.

**Methods:**

We sampled randomly 60 Cochrane reviews from 2008, which presented a result in its first meta-analysis with substantial heterogeneity (I^2 ^greater than 50%, i.e. more than 50% of the variation is due to heterogeneity rather than chance). We extracted information on choice of statistical model, how the authors had handled the heterogeneity, and assessed the methodological quality of the reviews in relation to this.

**Results:**

The distribution of heterogeneity was rather uniform in the whole I^2 ^interval, 50-100%. A fixed effect model was used in 33 reviews (55%), but there was no correlation between I^2 ^and choice of model (P = 0.79). We considered that 20 reviews (33%), 16 of which had used a fixed effect model, had major problems. The most common problems were: use of a fixed effect model and lack of rationale for choice of that model, lack of comment on even severe heterogeneity and of reservations and explanations of its likely causes. The problematic reviews had significantly fewer included trials than other reviews (4.3 vs. 8.0, P = 0.024). The problems became less pronounced with time, as those reviews that were most recently updated more often used a random effects model.

**Conclusion:**

One-third of Cochrane reviews with substantial heterogeneity had major problems in relation to their handling of heterogeneity. More attention is needed to this issue, as the problems we identified can be essential for the conclusions of the reviews.

## Background

Variability among individual study results in systematic reviews virtually always occurs. This is caused partly by random error (chance) and partly by systematic differences between the trials. The variation in the true effects is called heterogeneity. Its impact on meta-analyses can be assessed by I^2 ^that describes the percentage of the variability that is due to heterogeneity [1,2]. Values greater than 50% are - rather arbitrarily - considered substantial heterogeneity [1].

Strategies for addressing heterogeneity in systematic reviews include checking that the data extracted from the trial reports are correct, which may often not be the case [3]; omitting meta-analysis; conducting subgroup analysis or meta-regression; choosing a fixed effect or a random effects model [2]; changing the statistical metric, e.g. from a risk difference to a relative risk [4,5]; and excluding studies.

The fixed effect model assumes that all the included studies are estimating the same true effect. The variation in findings among studies is therefore due to chance [2]. Each study will be assigned a weight depending on the study's precision (within-trial variance) and an overall estimate can be calculated. Small studies will contribute relatively little to the outcome because they have less precision [6].

The random effects model assumes that the effects being estimated in the different studies are not identical, but follow a distribution. The confidence interval takes account of the additional uncertainty in the location of the mean of the systematically different effects in the different studies (this between-trial variance is added to the within-trial variance). Small studies will therefore contribute more to the average than in a fixed effect analysis, which is reasonable because the studies represent different true effects. Thus, when heterogeneity is present, the confidence interval around a random effects pooled estimate is wider than a confidence interval around a fixed effect pooled estimate [6].

Dealing with heterogeneity is often tricky, and there is only limited advice for authors on what to do, e.g. on when a particular model should be chosen for the other [7], or when the heterogeneity becomes too large for a meaningful meta-analysis.

An additional complexity is that the test for detecting heterogeneity has low power when the sample sizes are small or when few trials are included. For example, 11 trials give just 10 degrees of freedom, like a t-test on two groups of 6 people each does. There is also variation in practice as to which P-value demonstrates significant heterogeneity [2], but as the power of the test is so low, it is common to choose P = 0.10. It is important to be aware, however, that the choice of statistical model should not be based on the outcome of a test of heterogeneity [2].

The aim of our study was to investigate how authors address different degrees of substantial heterogeneity in meta-analyses in Cochrane reviews.

## Methods

We listed all Cochrane reviews from the Cochrane Database of Systematic Reviews 2008, Issue 1, which had at least one meta-analysis and where the first outcome in the first comparison involved all studies ('total'), and not only subgroups of studies ('subtotals'). We assumed that in most cases the first outcome in the first comparison would be the primary outcome and that in the remainder, it would still be important for the review.

**Figure 1:**
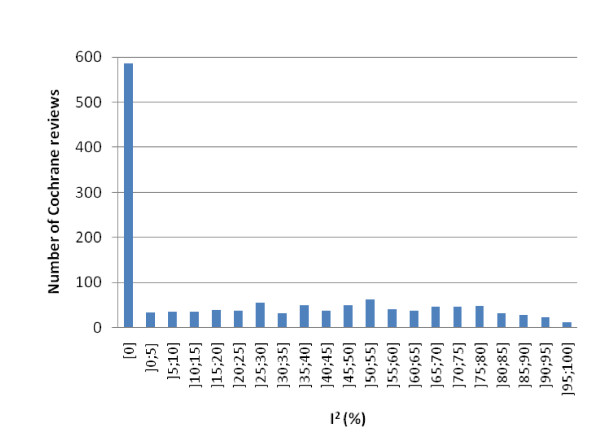
**Frequency distribution of Cochrane reviews in relation to I^2 ^for the first outcome in the first comparison**.

There were 3,385 Cochrane reviews, and of these, 2,354 (70%) had a least one meta-analysis, and 1,366 (40%) had a 'total' for the first outcome. Figure 1 shows that the distribution of I^2 ^for the 1,366 meta-analyses was rather uniform, apart from a decline in numbers of reviews with the most extreme degrees of heterogeneity and a large number of reviews in the group with I^2 ^of 0%. The latter result is expected since the calculation of I^2 ^gives many negative values, in which case I^2 ^is by definition set to zero [1].

Because of the relatively smooth distribution, we randomly selected 60 reviews with an I^2 ^of more than 50% for our study, using the random numbers generator in Excel. After having assessed the 60 reviews, it was clear that we had enough information to elucidate how authors address different degrees of substantial heterogeneity.

For every review, one observer (JBS) copied the relevant data into an Excel spreadsheet and a second observer (PCG) checked the data. Disagreements were few and were resolved by discussion. The extracted data were: i) The selected statistical model (random or fixed); ii) Any rationale for choosing the model; iii) The critical value for considering heterogeneity statistically significant; iv) Reservations about the results in relation to choice of model and comments on the heterogeneity; v) Attempts at explaining the heterogeneity narratively, e.g. different doses, populations, length of follow-up or quality of the included studies; vi) Attempts at addressing the heterogeneity statistically, e.g. by division of studies in subgroups, test for interaction, sensitivity analysis with omission of some studies, or meta-regression; this information was extracted from the Results section and in some cases directly from the graphs; vii) Point estimate and its P-value; the point estimate was also calculated with the alternative effect model, using the built-in facility for this in The Cochrane Library; viii) The P-value for the chi-square test for heterogeneity.

We assessed the overall methodological quality of the review based on whether the above points were addressed at all and focusing on if there were major problems in handling and interpretation of heterogeneity. We decided *a priori *that using a random effects model was a reasonable way of addressing substantial heterogeneity (unless there were special circumstances, as discussed below), and our assessments therefore focused mostly on those reviews where the authors had used a fixed effect model or where only one of the two models yielded a statistically significant estimate. We strived to be conservative in our judgments. If, for example, the authors had used a fixed effect model and gave the result of the heterogeneity test in the Results section, we interpreted this as a reservation about the result in relation to choice of model even if the authors provided no comments. Similarly, when a random effects model was chosen we interpreted this as a reservation.

We investigated whether the choice of model depended on the degree of heterogeneity, or on the P-value for the heterogeneity test. We did this because the reviews were produced at different times. Before the I^2 ^was developed, authors often relied on the P-value to identify heterogeneity [1].

## Results

### Choice of model in relation to degree of heterogeneity

**Figure 2:**
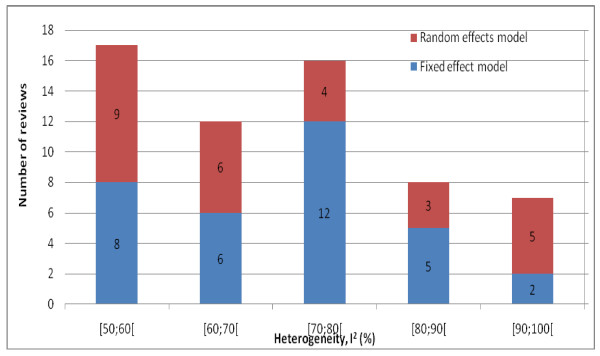
**Choice of model, reviews grouped by I^2^**.

Several of the 60 selected reviews had been published more than once. The oldest was most recently updated in 1996 and the newest in 2007 (median 2005). A fixed effect model was used in 33 reviews (55%), and a random effects model in 27 reviews (the Cochrane software does not allow mixed models [8]). There was no correlation between degree of heterogeneity and choice of model (Figure 2, P = 0.79, Mann-Whitney test for trend, with correction for ties), in fact the average I^2 ^was 71%, both for reviews using a fixed effect model and for those using a random effects model.

The authors selected the random effects model more often in the newest half of the reviews (updated later than 1 June 2005, Table 1), than in the oldest half. The same pattern was evident for the subgroup of reviews with marginally statistically significant heterogeneity (P-value between 0.05 and 0.10, P = 0.007, Fisher's exact test).

**Table 1:** Choice of model in relation to the P-value for the heterogeneity test.

	Newer reviews		Older reviews	
**P**	**Random**	**Fixed**	**Random**	**Fixed**

**< 0.0001**	6	0	2	6
**[0.0001;0.001[**	2	1	2	1
**[0.001;0.01[**	1	2	0	3
**[0.01;0.05[**	7	4	1	4
**[0.05;0.1[**	6	2	0	8
**> = 0.1**	0	0	0	2
**Total**	22	9	5	24

Newer reviews are those updated after 1 June 2005 (n = 31). P = 0.007 for those reviews where the heterogeneity test yielded a P-value between 0.05 and 0.10

### Significant effects in relation to choice of model

A significant effect estimate (P < 0.05) was presented in 34 reviews. For 6 of the 60 reviews, a significant result changed to a non-significant result when we applied the alternative model (discussed further below). For 5 reviews, a non-significant result became significant when we used the alternative model (Table 2). These 5 reviews had all used a random effects model and addressed heterogeneity this way; they were free of major methodological problems and will therefore not be discussed further.

**Table 2:** Results using the authors' model and the alternative model we applied

		Authors' model		
			
		Significant	Non-significant	Total
Alternative model	Significant	28	5	33
	Non-significant	6	21	27
	
Total		34	26	60

For 2 of the 6 reviews [9,10] where a significant result changed to a non-significant when we used a random effects model, the authors were cautious about their heterogeneous result and didn't base their conclusion on the significant finding they had obtained with a fixed effect model, which we consider a correct approach. One review was explicit about this: "*Substantial heterogeneity was also detected (p = 0.03, I^2 ^= 79%). Because of this, the result of this analysis should be interpreted with caution and not be considered a definitive statement" *[10].

The authors of the other 4 reviews were less cautious. One review [11] calculated mean differences instead of standardized mean differences, although the outcomes were measured on very different scales. Because of this error, both the means and the standard deviations differed by a factor of 10. This resulted in extreme heterogeneity (I^2 ^= 93%, P = 0.0002) despite very low power, as only two studies were included. In the methods section, the authors promised to use a random effects model in case of heterogeneity, but this was not done (and would not have solved the other problem).

In another review [12], the authors calculated the standardized mean difference both with a fixed effect model (1.07, 95% confidence interval 0.43 to 1.70) and a random effects model (1.74, -0.71 to 4.19). In the methods section, they stated they would use a random effects model if heterogeneity was present, which it was (I^2 ^= 89%, P = 0.002). With a random effects model, the result was not significant. They wrote that no definite conclusion could be made but added that there was reasonable evidence that cognitive therapy was beneficial in treating depression. We find this conclusion doubtful, given the data and their declared methods. In this example, the effect estimate calculated by the two models differs substantially due to a one small outlying study. Hence, the choice of model should have been considered and explained in detail.

Another review [13] used Peto's odds ratio (0.28, 0.11 to 0.73). Significant heterogeneity was present (I^2 ^= 59%, P = 0.05), and when using the ordinary odds ratio and a random effects model, the result became 0.28 (0.05 to 1.55). The authors concluded in the abstract that albumin showed a clear benefit at preventing severe ovarian hyperstimulation syndrome, although they were much more cautious in the main text.

The authors of the last review [14] reported that the P-value for heterogeneity was insignificant even though it was 0.07 and the power of the heterogeneity test was very low, as there were only 5 studies. They reported less mortality in the intervention group, relative risk 0.86 (0.74 to 1.00). With a random effects model the relative risk became 0.82 (0.57 to 1.18), with P = 0.30. The authors mentioned that heterogeneity was present and noted that one outlying trial had a very low mortality in the control group. The meta-analysis was driven by a big trial, which comprised 69% of the deaths and showed the same result as the pooled result, 0.86 (0.75 to 1.00). Even so, we find it pretty bold that the authors believed in a result with borderline significance (P = 0.05), and only when using the fixed effect model, with so much heterogeneity, and with unexplained discrepancies between the results of the trials.

### Cautions about the heterogeneity

In our judgment, 40 of the 60 reviews were devoid of major problems in relation to their handling of heterogeneity (Table 3). However, only 27 reviews (45%) gave a rationale for choice of statistical model. Our overall judgment of methodological quality was not related to I^2 ^(P = 0.26, Mann-Whitney test for trend corrected for ties, grouping I^2 ^into intervals of 10%). However, the unproblematic reviews contained more studies than problematic reviews, 8.0 versus 4.3, on average (P = 0.024, student t-test, two-tailed).

**Table 3:** Our assessments of the 60 reviews in relation to their handling of heterogeneity

	Reviews	Per cent
**Overall acceptable methodological quality**	40	(67%)
**Rationale given for choice of model**	27	(45%)
**Valid reservations against results**	36	(60%)
**Explanation of causes of heterogeneity**	40	(67%)
**Explanation reasonable**	36	(60%)
**Heterogeneity addressed statistically in the analysis**	39	(65%)

**Figure 3:**
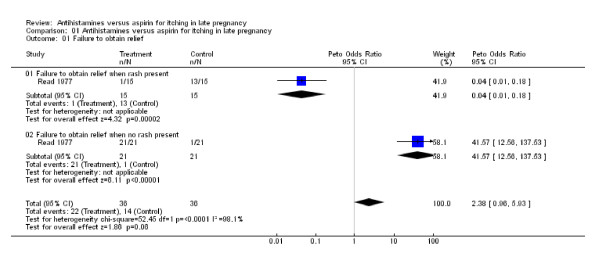
**Extremely diverging results**. Both results originate from the same study [21].

Sixteen of the 20 problematic reviews had chosen a fixed effect model. There can be plausible reasons for this, even in cases with substantial heterogeneity, but authors should then explain what they are. Eight of these 20 reviews had no explanations or reservations and did not address the heterogeneity statistically [15-22]. One review [21] had only included one study, but the patients were split into two subgroups, according to whether they had a rash. Both results were significant, but one showed harm and the other benefit (Figure 3). Combining such results is inappropriate and doesn't represent today's standards of Cochrane reviews (the review was last updated in 1996).

Five other reviews used Peto's odds ratio [23,24,13,18,19], and three reviews didn't follow the analysis plan that was set out in the methods section [12,25,26], which included using a random effects model or omitting meta-analysis in case of heterogeneity, and there was no explanation why. Three other reviews paid no attention to the heterogeneity and didn't discuss it, even though the P-value was between 0.05 and 0.10 [27,14,28]. An additional review described the heterogeneity (I^2 ^= 71%, P = 0.06) but ignored it due to "lack of stability of the known tests" [29], which is not a valid reason for ignoring heterogeneity. In another review, the authors divided the analysis into subgroups because they had found heterogeneity, but although the consequence was that the chi-square test for heterogeneity was no longer significant due to loss of power, the I^2 ^actually *increased*, which the authors failed to comment on [30]. In yet another review, which was discussed above, the authors pooled two risk scores measured on different scales that varied by a factor of ten [11]. The complete list of included reviews can be found on the web http://sites.google.com/site/dealingwithheterogeneity/.

## Discussion

**Figure 4:**
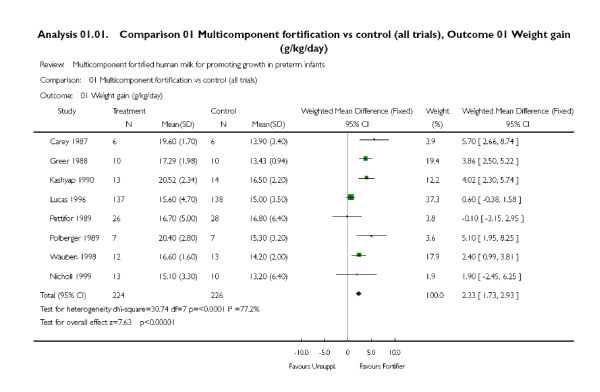
**Results analysed using a fixed effect model, which gives more weight to the only large study **[22].

It can be challenging to choose the most appropriate model for meta-analysis, as there are pros and cons with both models [2]. It is pretty clear that the larger the heterogeneity, the harder it is to defend choosing a fixed effect model, as different studies cannot be assumed to provide estimates of a common, true effect. However, one also needs to consider that a random effects model may apply too much weight to small studies, which are often poorly done and biased. An example is shown in Figure 4, where it appeared reasonable that the authors used a fixed effect model despite pronounced heterogeneity, as it gives more weight to the only large study, which, moreover, had a result that was closer to no effect than most other studies. It is also possible that the studies were so different that they should not have been combined in a meta-analysis. Only a closer look at the review, and possibly also at the individual studies, can elucidate this, and even then, researchers may disagree what would be the most appropriate approach.

It is also important to consider that the fixed effect model only allows an inference about the studies included in the meta-analysis, whereas the random effects model allows an inference about the mean effect in a hypothetical population of studies if we can assume that the studies included in the meta-analysis constitute a random selection of studies from this hypothetical population.

The random effects model is more conservative than the fixed effect model in the sense that the confidence interval is broader, but sometimes the point estimate is farther from the null and the P value for the pooled effect smaller than with a fixed effect model [31].

When using a random effects model, the between-study variance needs to be calculated, but if there are few studies, this cannot be calculated with any precision, and a fixed effect model is therefore sometimes used in this situation [6].

It was surprising that we did not find a relation between the degree of heterogeneity and the choice of model. Some Cochrane groups instruct their authors to routinely use a fixed effect model, although few statisticians would find such blanket recommendations reasonable. Furthermore, in all types of research, authors should change their planned analysis and explain why if it would not be sensible.

**Figure 5:**
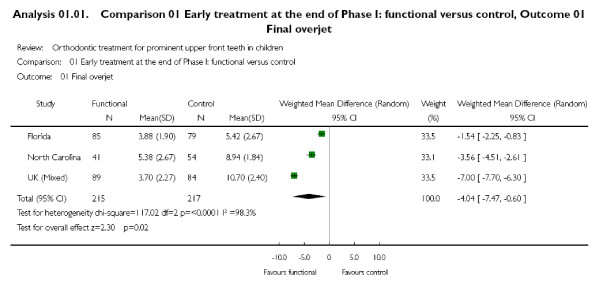
**Example of extreme heterogeneity **[20].

Readers might be more willing to accept the results if they are robust to both types of analyses, but we found only one example of this approach [32]. Authors should also consider the possibility of abstaining from meta-analysis and explore the reasons for the heterogeneity instead, and we identified several reviews where this might have been better. For example, one review with extreme heterogeneity (I^2 ^= 98%, P < 0.0001) pooled three trials with a random effects model although *none *of them had overlapping confidence intervals (Figure 5)[20].

Although our sample consisted only of reviews with substantial heterogeneity, about a third of the authors had not paid any attention to it. This omission was quite uniform over the spectrum of I^2 ^values, and it might therefore partly reflect the well-known lack of statistical skills among authors of medical research papers [33-35]. However, as authors are recommended to routinely assess whether the results are consistent across studies [2], and what the likely causes are if they are not, they could do better even without having access to statistical expertise. Cochrane review groups could also do better, as they are required to have access to statistical expertise [2]. Recently, summary of findings tables were introduced in Cochrane reviews as part of GRADEprofiler, where the authors are asked to assess the quality of the body of evidence. This includes assessing the likelihood that the pooled estimate for each outcome is free from bias [2], and a judgment related to the degree of heterogeneity.

Reviews that were devoid of major problems had included more trials than those with problems. The likely reason for this is that authors are usually too influenced by whether or not a P-value is significant and often do not take into account, or do not know, that P-values depend on the number of trials. When fewer trials are included, it is harder to identify heterogeneity using a chi-square test. This test is therefore not the recommended way to investigate heterogeneity [1]. I^2 ^is more sensitive but with few included trials there is a small risk of false positives.

### Limitations

Our sampling method precludes us from drawing general conclusions about the quality of Cochrane reviews in relation to heterogeneity. As we sampled meta-analyses, we did not assess how often the authors had abstained from pooling the results because of heterogeneity, which would have been an arduous task, given our total sample of 3,385 reviews.

The most important assessment - whether a review was devoid of major problems related to heterogeneity - was not as thoroughly specified in our protocol as we would have wished. It would not have been possible to specify in advance rigid rules because of the great diversity in handling and reporting heterogeneity. We have compensated for this limitation by describing the problematic reviews we encountered. More strict criteria could be used in future studies based on our findings.

In a few reviews, our outcome was not a primary one, which could be the reason that the heterogeneity was not addressed. On the other hand, these reviews tended to not address heterogeneity at all, for any outcomes.

We specified in our protocol that we wanted to investigate to which extent the point estimates and the confidence interval varied when a different model was chosen, but decided to focus on reviews where the result changed from significant to nonsignificant and vice versa.

Some of our analyses were exploratory. During data extraction, we decided to investigate if there was a relation between the choice of model and the P-value for heterogeneity, and we couldn't help noticing that the reviews we judged to be most problematic also tended to be those that had included fewest trials.

It is known that I^2 ^increases when the sizes of the included studies increase and alternative measures of heterogeneity have been suggested [36]. However, the problematic reviews identified in our study included very few trials and relatively few participants. When there are only few included trials there is a small risk of I^2 ^above 50% even though no heterogeneity is present.

### Other studies of heterogeneity

In the early years of the Cochrane Collaboration, randomly selected Cochrane reviews were assessed by two different observers, and 29% were judged to have major problems [37], but these concerned other issues than heterogeneity. In another study of Cochrane reviews, heterogeneity, defined as P < 0.10, was identified in 34 out of 86 meta-analyses, and in 12 of the 34 meta-analyses, heterogeneity was not addressed [38]. In 2002, Higgins et al. [7] investigated the newest Cochrane reviews and tested if heterogeneity was present, and collected information about choice of model and subgroup analyses. The study compared the protocol to the review and identified problems concerning choice of statistical model and problems with conducting subgroup analyses, as there were often too few included trials.

## Conclusion

One-third of Cochrane reviews with substantial heterogeneity in the first reported outcome had major problems in relation to their handling of heterogeneity. These consisted mainly of the use of a fixed effect model without an explicit rationale for choice of that model, and lack of reservations and explanations of the likely causes of the heterogeneity. These problems became less pronounced with time, as those reviews that were most recently updated much more often used a random effects model. More attention is needed to this issue, as the problems we identified can be essential for the conclusions of the reviews.

## Competing interests

We all work at a Cochrane Centre.

## Authors' contributions

JBS and PCG designed the study, carried out the statistical analysis and analyzed the data. RM extracted the list of meta-analysis and revised the manuscript. JS extracted data from each meta-analysis later verified by PCG. All authors read and approved the final manuscript.

## Pre-publication history

The pre-publication history for this paper can be accessed here:

http://www.biomedcentral.com/1471-2288/11/22/prepub
